# Characterization of non-standard viral genomes during arenavirus infections identifies prominent S RNA intergenic region deletions

**DOI:** 10.1128/mbio.01612-24

**Published:** 2024-09-11

**Authors:** Matthew Hackbart, Carolina B. López

**Affiliations:** 1 Department of Molecular Microbiology and Center for Women Infectious Disease Research, Washington University School of Medicine, St. Louis, Missouri, USA; Boston University Chobanian & Avedisian School of Medicine, Boston, Massachusetts, USA; Boston University, Boston, Massachusetts, USA

**Keywords:** arenavirus, non-standard viral genomes, deletions, lymphocytic choriomeningitis virus, viral interference

## Abstract

Arenaviruses, a family of negative-sense RNA viruses spread by rodents, are a leading cause of severe hemorrhagic fever in humans. Due to a paucity of antivirals and vaccines for arenaviruses, there is a need to identify new mechanisms for interfering with arenavirus replication. In several negative-sense RNA viruses, natural viral interference results from the production of non-standard viral genomes (nsVGs) that activate the innate immune system and/or compete for essential viral products. Although it is well established that arenaviruses produce strong interfering activities, it is unknown if they produce interfering nsVGs. Here, we show that arenaviruses produce deletions within the intergenic region of their small (S) RNA genome, and these deletions inhibit viral glycoprotein production during minigenome replication. S RNA deletions are more abundant when arenaviruses are grown in high-interfering conditions and are associated with reduced viral replication. Overall, we found that arenaviruses produce internal deletions within the S RNA intergenic region that are capable of decreasing glycoprotein production. These natural arenavirus interfering molecules provide a new target for the generation of therapeutics against arenaviruses.

**IMPORTANCE:**

Arenaviruses are hemorrhagic fever-causing pathogens that infect millions of people a year. There are currently no approved antivirals that target arenaviruses, and understanding natural mechanisms that inhibit arenavirus replication is crucial for the development of effective therapeutics. Here, we identified multiple deletions within arenavirus genomes that remove major replicative elements of the viral genomes. We show that deletions that remove the intergenic region of the viral genome can prevent viral protein production. These deletions were found in all arenaviruses tested in this study representing a mechanism that could be harnessed for the development of antivirals that broadly target the arenavirus family.

## INTRODUCTION

Arenaviruses are negative-stranded, bi-segmented RNA viruses and are major causative agents of hemorrhagic fever. For example, Lassa virus causes 2 million cases and 5,000–10,000 deaths annually (1–4). Although arenaviruses are endemic in Africa and South America, there is only one approved vaccine that targets Junin virus (JUNV) and there are no targeted antivirals (5). Discovering new mechanisms for attenuating arenavirus infections is imperative for the development of antivirals and vaccines.

**Fig 1:**
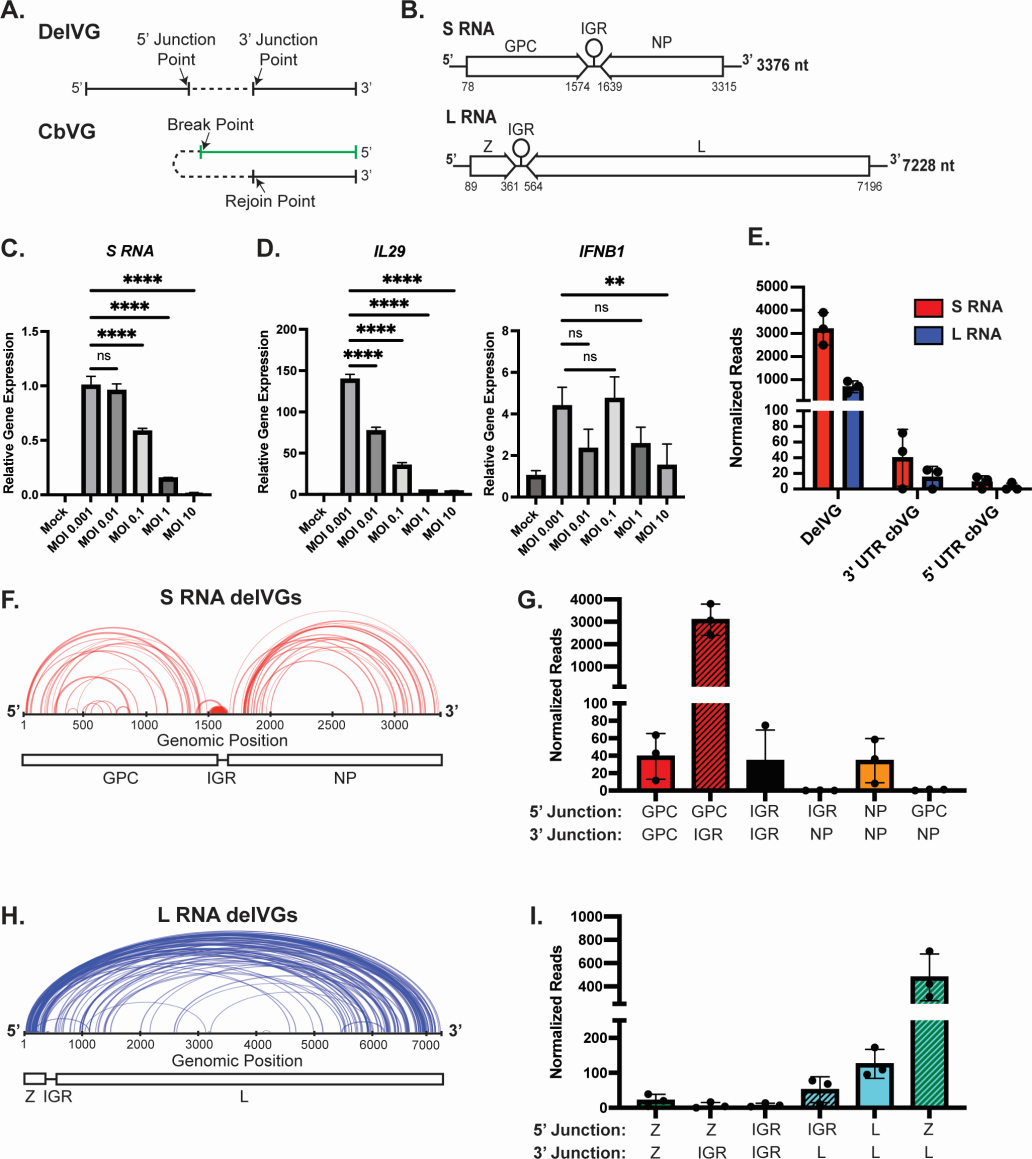
The most consistent nsVGs produced during lymphocytic choriomeningitis virus (LCMV) infection are small (S) RNA delVGs. (**A**) Schematic of a delVG with the junction points indicated by arrows and a cbVG with the break and rejoin point indicated by arrows. (B) Diagram of the LCMV genomes. GPC, glycoprotein complex; IGR, intergenic region; NP, nucleoprotein; Z, matrix protein; L, polymerase. (C and D) A549 cells were infected with LCMV at indicated multiplicities of infection (MOIs). At 48 hours post-infection (hpi), cellular RNA was collected and quantitative polymerase chain reaction (qPCR) was performed for (C) viral S RNA or (D) *IL29* or *IFNB1* mRNA. Gene expression was normalized to a housekeeping index of *β-actin* and *GAPDH*. qPCR data are representative of three independent repeats with similar results. One-way ANOVA was performed for statistical analysis. ns, *P* > 0.05; **, *P* < 0.01; ****, *P* < 0.0001. (**E–I**) A549 cells were infected with LCMV Armstrong at an MOI of 0.1. RNA was collected at 48 hpi, sequenced, and analyzed using the VODKA2 pipeline. Reads were normalized as nsVG reads per 1 million viral reads. (E) Quantification of nsVG species for the S RNA (red) and L RNA (blue). (**F**) Representation of 5′ junction and 3′ junction points of individual S RNA delVGs. Each arc represents an individual delVG species with the width of the arc proportional to the normalized reads detected per delVG species. Data are representative of three independent sequencing experiments with similar results. (**G**) Quantification of S RNA delVGs that contain 5′ and 3′ junctions in indicated genomic regions. Data are from three independent sequencing experiments. (**H**) Representation of 5′ junction and 3′ junction points of individual large (L) RNA delVGs. Each arc represents an individual delVG species with the width of the arc proportional to the normalized reads detected per delVG species. Data are representative of three independent sequencing experiments with similar results. (**I**) Quantification of L RNA delVGs that contain 5′ and 3′ junctions in indicated genomic regions. Data are from three independent sequencing experiments.

One natural mechanism for inhibiting viral replication results from viral interference. Viral interference occurs when a virus inhibits its own replication or the replication of another virus through the induction of innate immune responses, such as interferons, modulation of cellular receptors, inhibition of viral protein translation, or competition for essential viral proteins (6–9). Viral interference during negative-sense RNA virus infections is associated with the production of non-standard viral genomes (nsVGs), also known as defective viral genomes. nsVGs can be deletion viral genomes (delVGs) or copy-back viral genomes (cbVGs) depending on the nature of the genomic rearrangement (Fig. 1A). For example, paramyxoviruses will produce cbVGs that can compete for viral polymerases, activate host innate immune sensors, and drive persistent infections (7, 8).

Arenaviruses produce two genomic RNA species: the large (L) RNA and the small (S) RNA. In an ambi-sense fashion, the L RNA encodes a matrix (Z) protein and a polymerase (L) protein, while the S RNA encodes the glycoprotein complex (GPC) and a nucleoprotein (NP) (Fig. 1B) (10–12). The S RNA and L RNA each have an intergenic region (IGR) in between the genes coding for viral proteins. Viral mRNA is produced from the genomic RNA and contains each individual gene and the IGR. The IGRs have RNA stem loops that function as transcription termination sites for the generation of the viral mRNA and are involved in the virus assembly and budding (13). Arenaviruses that contain genomes with switched IGRs (e.g., L RNA containing the S RNA IGR) are severely attenuated *in vivo* (14–18). In addition, arenaviruses can undergo widespread recombination events, such as the insertion of multiple IGRs or duplication of gene segments (19), but it is unknown if these recombination events are related to viral interference.

Studies with the arenavirus lymphocytic choriomeningitis virus (LCMV) have provided evidence for the presence of nsVGs and viral interference during infection, including the following: (i) LCMV can produce high levels of defective interfering particles, which block viral replication and spread of LCMV infection (10, 20–23). (ii) Unique RNA species that do not match the viral genomes or mRNA are detectable by northern blot during persistent LCMV infection (24). (iii) LCMV infections can produce viral RNA containing small deletions of the genomic ends (25), which may be products of the cap-snatching activity of the viral polymerase (26). (iv) Arenavirus vaccine candidates produce differential viral RNA species that contain large internal deletions (14).

With the goal of further understanding arenavirus interference, we sought to identify nsVG species produced naturally during arenavirus infection and determine if and how these nsVGs can interfere with virus replication. We show that LCMV produces internal delVGs in the S RNA IGR that are associated with decreased viral replication. Utilizing RNA sequencing and our bioinformatics pipeline, VODKA2, we found that LCMV, JUNV, and Parana virus (PARV) produce cbVGs and delVGs in both the S and L RNAs. We observed that the most abundant delVGs occur over the S RNA IGR and then detected these specific delVGs with RNA fluorescent *in situ* hybridization. To determine which of the nsVGs are associated with viral interference, we generated two viral stocks: one virus passaged at the standard growth condition and one virus passaged at high multiplicity of infection (MOI) to enhance interfering RNAs. We found that S RNA IGR-delVGs are increased in abundance in the viral stock with viral interference, correlating IGR-delVGs with the inhibition of infectious particle production. Using a minigenome (MG) system to determine the mechanism of viral interference, we found that the IGR-delVGs decrease the production of the viral glycoprotein. Overall, we found novel delVGs in the LCMV S RNA IGR that are capable of inhibiting viral replication through the inhibition of viral glycoprotein production.

## RESULTS

### LCMV predominantly produces delVGs

To determine if naturally produced nsVGs can inhibit arenavirus replication, we first characterized the types and quantities of nsVGs that are produced during arenavirus infections. To start, we infected BHK21 cells at an MOI of 0.001 for 72 hours to prepare a stock of LCMV Armstrong 53b (LCMV-Arm) that was capable of replicating to high viral titers according to published protocols (27). To test if interference occurred, we infected A549 lung epithelial cells with LCMV-Arm at increasing MOIs. We observed a decrease in viral RNA at 48 hours post-infection (hpi) as the MOI increased, despite no indication of cell death (Fig. 1C; Fig. S1A and B). Differently from interference observed in other negative-sense RNA viruses, including paramyxoviruses and pneumoviruses (28, 29), the reduction of LCMV RNA did not associate with the increased expression of type III interferon (*IL29*) or type I interferon (*IFNB1*) (Fig. 1D).

As interference in RNA viruses is generally associated with the presence of nsVGs, we next looked for the production of nsVG species 48 hpi at an MOI of 0.1, when the virus produces high levels of viral RNA but shows the phenotype of viral interference. We used next-generation sequencing to identify nsVGs from LCMV-infected cells using our custom bioinformatics pipeline, VODKA2 (30). We defined specific species of delVGs by the position of the 5′ nucleotide of the junction and the 3′ nucleotide of the junction (Fig. 1A). Since LCMV is an ambi-sense virus, we also analyzed cbVGs that contained either the 3′ untranslated region (UTR) or 5′ UTR of the viral genome. The cbVGs species are defined by the theoretical break and rejoin points of the recombination event as previously described (Fig. 1A) (7, 8). We found that LCMV-Arm produces cbVGs containing the 3′ UTR or 5′ UTR, but a majority of the nsVGs found were delVGs (Fig. 1E; Table S1 to S3). Both the S RNA and L RNA genomic segments produced delVGs with distinct distributions of junction points (Fig. 1F and H). The S RNA had one major set of abundant delVGs that removes the end of the GPC gene and part of the IGR. The S RNA also contains minor deletions that removed internal portions of the GPC gene, the IGR, or the NP gene (Fig. 1F and G). Most of the L RNA delVG species contain 5′ junction points occurring in the first 500 nucleotides within the Z gene and 3′ junction points occurring after 5,000 nucleotides of the genome, thereby deleting most of the L polymerase gene (Fig. 1H and I). Minor L RNA delVGs also occur within the L gene and the IGR-L segment. Interestingly, both S RNA and L RNA delVGs tend to remove the IGRs of the genomic RNAs, which are necessary for viral replication (13). Unlike the delVGs, the cbVG species are more evenly distributed throughout the genome (Fig. S2A and B). We detected S RNA delVGs in similar abundance in both cellular and supernatant RNA (Fig. S2C), but increased levels of L RNA delVGs in the supernatant of infected cells (Fig. S2D). The presence of the delVGs in the supernatant RNA suggests that these delVGs can be packaged and transmitted during infection.

**Fig 2:**
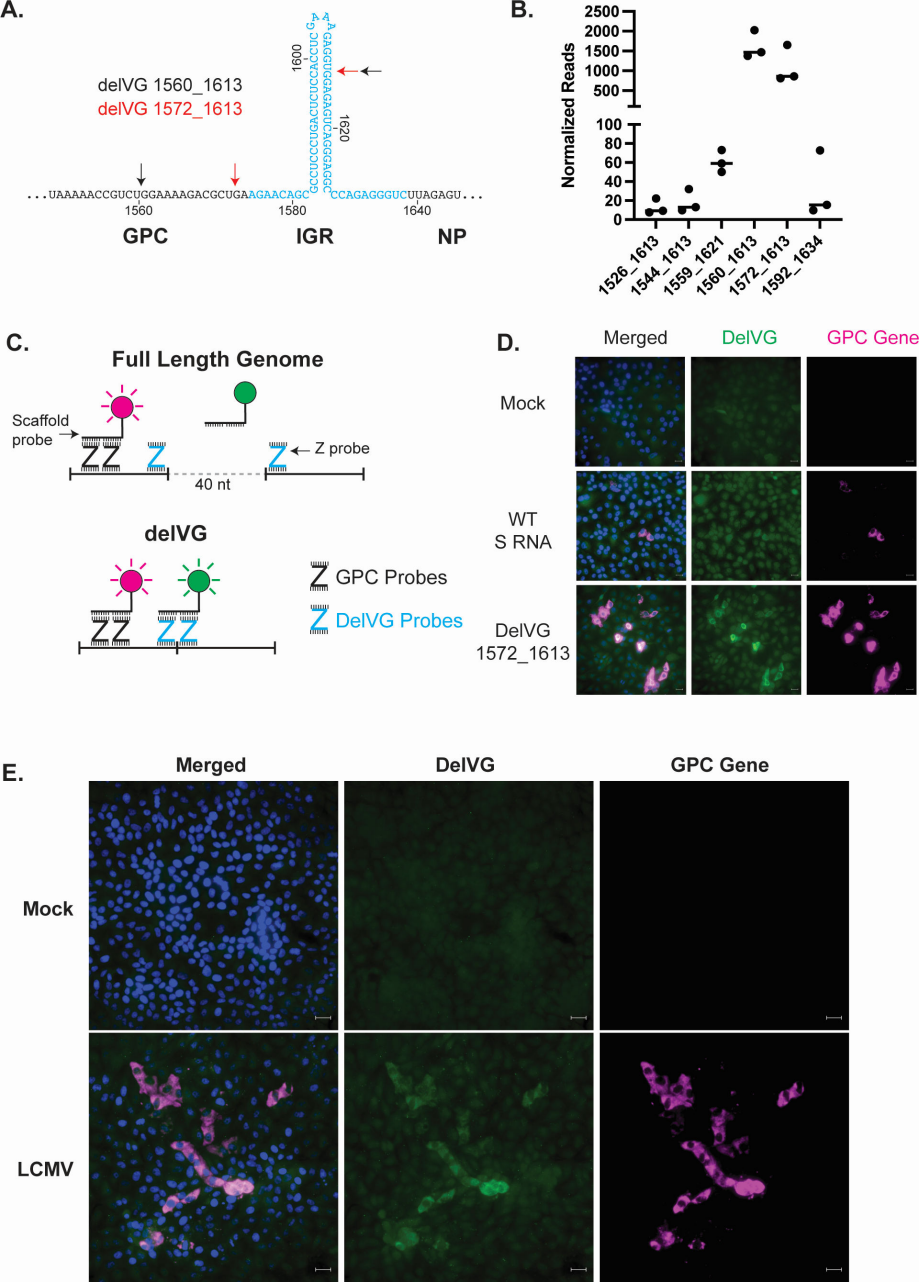
The most abundant S RNA delVGs delete segments within the GPC-IGR. (**A**) Locations of abundant S RNA IGR-delVGs. The IGR is highlighted in blue. Black arrows indicate the junction points for delVG 1560_1613, and red arrows indicate the junction points for delVG 1572_1613. (**B**) Quantification of the individual IGR-delVGs. Data are from three independent sequencing experiments. (**C**) Schematic for RNAscope with Z probes binding to the wild-type or delVG genomes. GPC gene probes produce a fluorescent signal (magenta) upon binding to the wild-type and delVG RNAs, whereas the delVG probes only produce a fluorescent signal (green) when binding to the delVG RNA. (**D**) Vero E6 cells were transfected with either plasmids encoding the S RNA or delVG 1572_1613 minigenomes. After 24 hours, cells were fixed, processed by RNAscope, and stained for nuclei (blue), delVG 1572_1613 (green), and GPC gene (magenta). The scale bar is 20 µm. (**E**) Vero E6 cells were infected with LCMV Armstrong at an MOI of 0.1. At 48 hpi, images were processed by RNAscope and stained for nuclei (blue), delVG 1572_1613 (green), and GPC gene (magenta). Scale bars are 20 µm. Images are representative of three independent imaging experiments.

The most abundant delVG species detected by RNA-Seq were deletions in the S RNA that contain 5′ junction points within the end of the GPC gene and 3′ junction points within the IGR stem-loop structure (Fig. 2A; Table S4). While the 5′ junction points varied, the most consistent 3′ junction point was around nucleotide 1613 in the IGR. The most abundant of these S RNA IGRs were IGR-delVG 1560_1613 and IGR-delVG 1572_1613, each having about 1,500 reads per 1 million viral reads detected (Fig. 2B). To test if the IGR-delVGs were specific to LCMV-Arm, we sequenced a stock of LCMV-Clone 13 (LCMV-Cl13) and found the dominant delVG species were also delVG 1560_1613 and delVG 1572_1613 (Fig. S3A and B). The IGR-delVGs were also detected by reverse transcription and polymerase chain reaction (PCR) over the LCMV-Arm S RNA IGR (Fig. S4A; Table S5).

### Major S RNA delVGs can be detected in infected cells using a PCR-independent strategy

As the IGRs play critical roles during viral replication, the abundance of IGR deletions prompted us to hypothesize that IGR-delVGs generated during virus replication are involved in LCMV interference. However, during the validation of our RNA-seq analysis pipeline, we learned that the IGR-delVGs can be detected from an *in vitro-*transcribed RNA that contained the GPC gene, IGR, and an mCherry gene replacing the NP gene (Fig. S4B and C). Detection of IGR-delVGs in the absence of viral replication proteins suggests that IGR-delVGs can be generated by either the T7 RNA polymerase, RT enzyme, or subsequent PCRs in the RNA-seq library preparation, and we needed to validate their presence during infection using T7 and RT-PCR independent methods. Utilizing direct RNA sequencing with Nanopore technology, we confirmed the presence of the IGR-delVGs and determined that the GPC-IGR region was preferentially deleted, similar to what we observed with the analysis of short-read sequencing (Fig. S4D and E).

Additionally, we confirmed the presence of the delVGs by utilizing Basecope Technology (ACD Biosystems), an RNA fluorescent *in situ* hybridization (FISH) assay that utilizes paired DNA “Z”-probes and amplifying scaffolding probes to produce a detectable fluorescent signal. Specifically, we used probes to detect the S RNA GPC gene and the delVG 1572_1613. The GPC probes, which serve as a marker for viral RNA, bind to the genome allowing the scaffolding probe to bind horseradish peroxidase and a fluorescent signal to be amplified with tyramide signal amplification (Fig. 2C). In the full-length standard viral genomes, the delVG Z-probes are separated by 40 nucleotides, so the scaffolding probes cannot efficiently bind to the two delVG Z-probes. However, when delVG 1572_1613 is generated, the 40 nucleotides are removed, and the Z-probes are then able to bind to the scaffolding probe, producing a fluorescent signal (Fig. 2C). To test this assay, we transfected plasmids that transcribed either the wild-type GPC gene or the delVG RNA into Vero E6 cells. While the RNA FISH probe against the GPC gene was detected for both transfections, the delVG probe set only produced a fluorescent signal when the delVG RNA was present (Fig. 2D). During viral infection, the delVG RNA was detectable in a subset of infected cells, confirming that delVG 1572_1613 is produced during LCMV infection (Fig. 2E).

### High levels of S RNA IGR-delVGs are found in high-interfering infections

**Fig 3:**
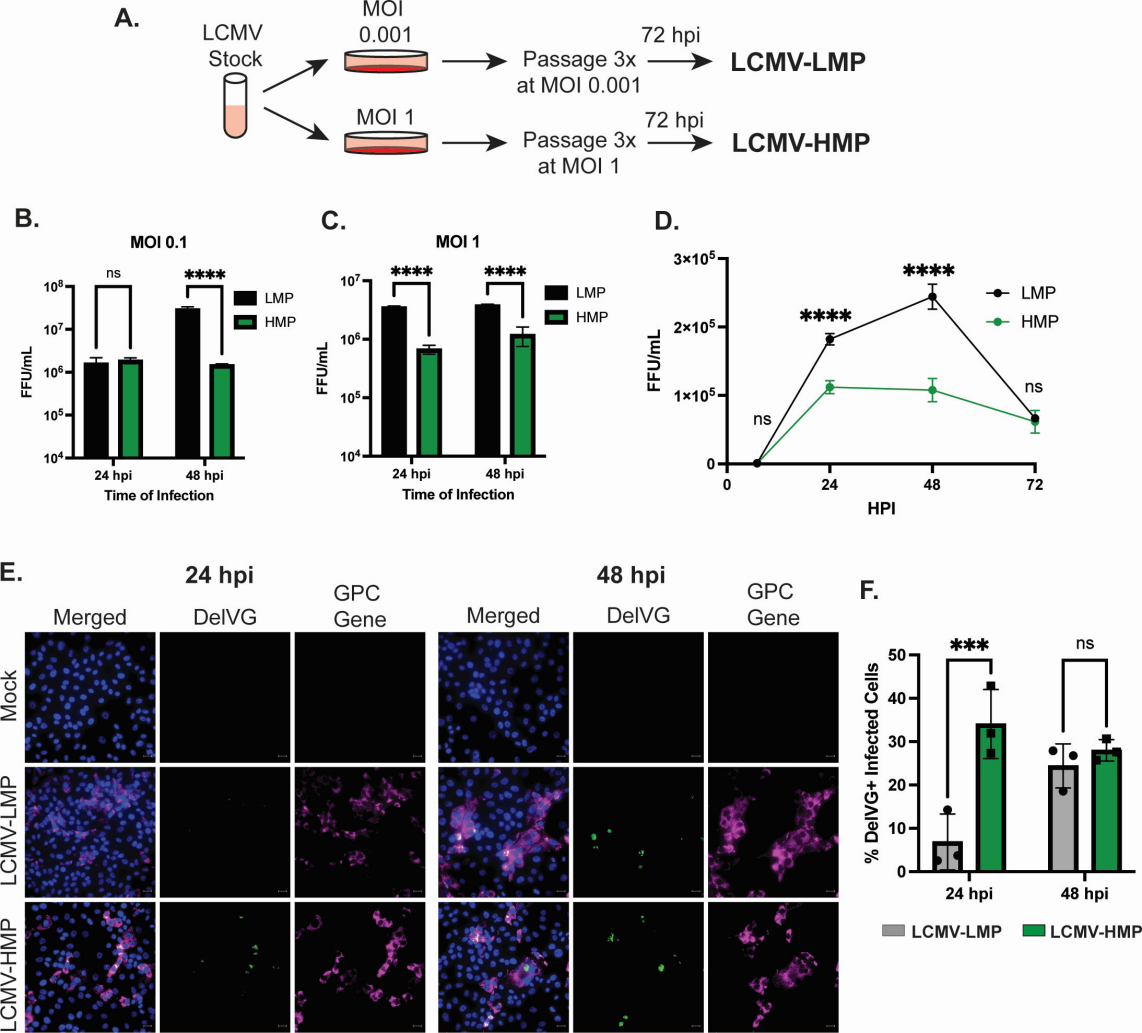
S RNA IGR-delVGs are increased during LCMV interference. (**A**) Strategy for the generation of LCMV stocks grown at the standard MOI of 0.001 (LCMV-LMP) and high MOI of 1 (LCMV-HMP). (B and C) BHK21 cells were infected with either LCMV-LMP or LCMV-HMP at an MOI of (B) 0.1 or (C) 1. The cellular supernatant was collected at indicated timepoints, and focus-forming units (FFU) were quantified. Data are from three biological replicate infections. Two-way ANOVA was performed for statistical analysis. ns, *P* > 0.05; ****, *P* < 0.0001. (**D**) Vero cells were infected with either LCMV-LMP or LCMV-HMP at an MOI of 1. The cellular supernatant was collected at indicated timepoints and FFU were quantified. Data are from three biological replicate infections. Two-way ANOVA was performed for statistical analysis. ns, *P* > 0.05; ****, *P* < 0.0001. (**E**) Vero cells were infected with either LCMV-LMP or LCMV-HMP at an MOI of 1. Cells were processed at 24 or 48 hpi with RNAscope for the detection of nuclei (blue), delVG 1572_1613 (green), and GPC gene (magenta). The scale bar is 20 µm. Images are representative of three independent imaging experiments. (**F**) Quantification of the percentage of GPC gene-positive cells that were delVG-positive. Two-way ANOVA was performed for statistical analysis. ns, *P* > 0.05; ***, *P* < 0.01. Data are from three independent imaging experiments with 200 cells quantified per condition.

We next tested if IGR-delVGs are enhanced in infections with LCMV stocks that have high-interfering activity. When comparing infections with increased MOIs, we observed an increase in delVG 1572_1613 at higher MOIs (Fig. S1C and D). To compare viral interference during infections at the same MOI, we generated different stocks of LCMV grown by either low MOI passaging (LCMV-LMP) or high MOI passaging (LCMV-HMP) (Fig. 3A). As expected, subsequent infection in BHK21 cells showed a decreased production of infectious particles during LCMV-HMP infection compared to LCMV-LMP (Fig. 3B and C). This interference did not associate with an enhanced early antiviral interferon response during the infection of A549 cells (Fig. S5A through C). Upon next-generation sequencing and VODKA2 analysis of the passaged stock supernatants, we discovered that the LCMV-HMP stock had an increased abundance of the IGR-delVGs, but no increase in other nsVGs (Fig. S6A and B). To determine if the increase in delVGs was a product of the higher MOI for the LCMV-HMP stock, we determined if LCMV-HMP had increased IGR-DelVGs during subsequent infections at an identical MOI. Upon the infection of Vero E6 cells with either the LCMV-LMP or LCMV-HMP, we again observed interference with the production of infectious particles during LCMV-HMP infection (Fig. 3D). Importantly, LCMV-HMP had increased abundance of IGR-delVG-positive cells at 24 hpi (Fig. 3E and F), while the LCMV-LMP infection eventually produced IGR-delVG-positive cells at 48 hpi. While we detected delVG 1572_1613 in up to 30% of infected cells at 48 hpi, the RNA probes used only targeted one species of S RNA delVG, and other delVGs are also highly detected by sequencing (ex. delVG 1560_1613). Additionally, the probes do not detect L RNA delVGs. We also observed an increase in delVG 1572_1613 at 24 hpi during high MOI infection by qPCR (Fig. S5D). These data indicate that LCMV with increased IGR-delVGs exhibits a higher level of viral interference without enhancing the activation of the interferon pathway. Interestingly, the presence of delVG-positive cells (Fig. 3E) precedes the reduction of infectious particles released from the cells (Fig. 3D) with delVG detected at 24 hpi with LCMV-HMP, prior to the decreased viral titer observed at 48 hpi. Similarly, delVG presence at 48 hpi with LCMV-LMP preceded the decrease in viral titer observed at 72 hpi. We did not observe cell death associated with the decrease in infectious particles, so we speculate that the delayed inhibition may be due to the packaging of the delVGs.

### The S RNA IGR-delVGs reduce protein production during minigenome replication

The arenavirus IGRs were previously shown to function as transcription termination sequences during the production of viral mRNAs. Minigenome replication systems that replicate the S RNA genome by co-expression with the NP and L proteins show that complete deletion of the S RNA IGR led to a loss of viral mRNAs and a decrease in viral protein production (13). Therefore, we hypothesized that our observed IGR-delVGs similarly inhibit the production of viral mRNA and protein production.

**Fig 4:**
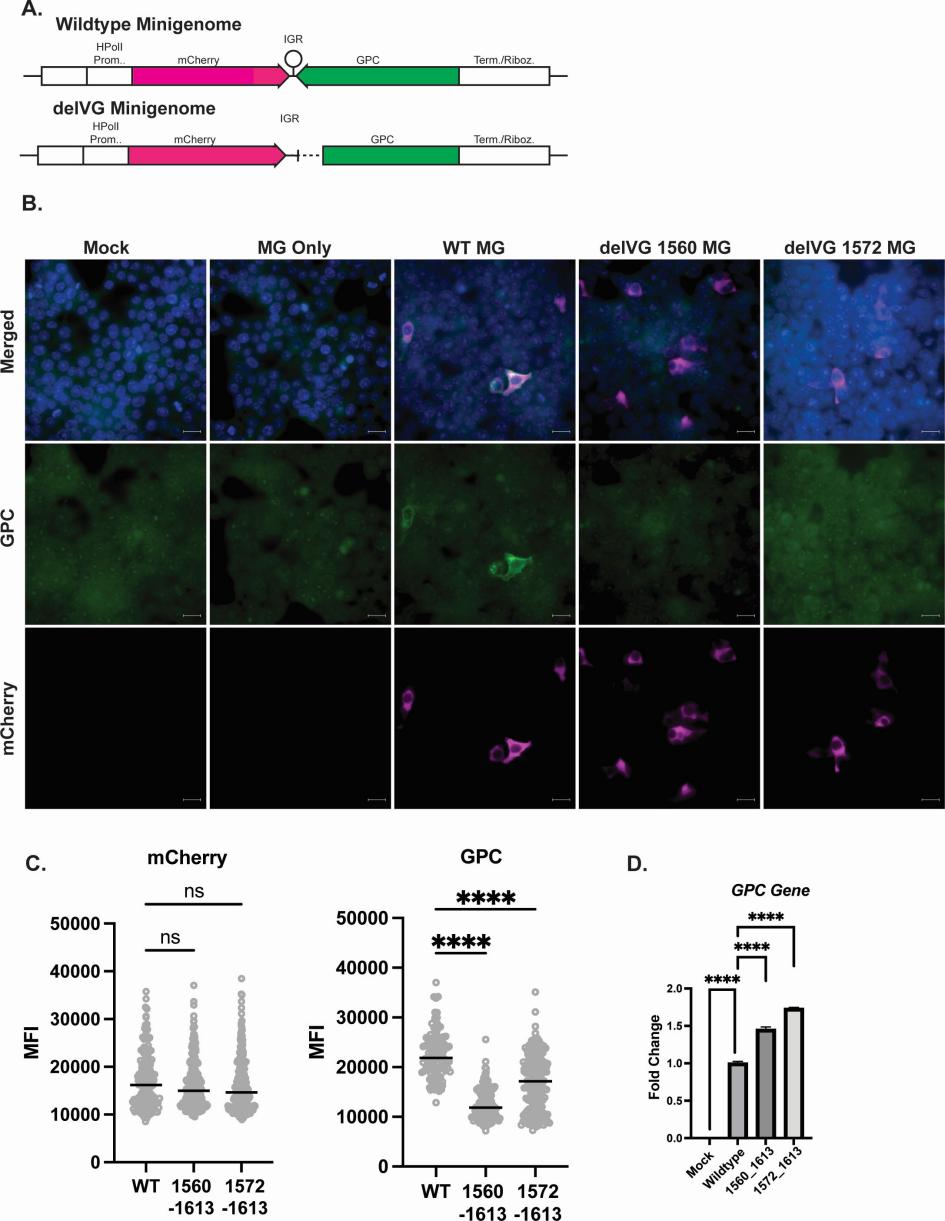
S RNA IGR-delVGs decrease viral protein production. (**A**) Diagram of LCMV MGs that contain the human PolI promoter followed by an mCherry gene, the IGR, the glycoprotein complex gene, and a termination/ribozyme sequence. The delVG minigenomes contain either the 1560_1613 or 1572_1613 deletions. (**B**) Vero E6 cells were co-transfected with the MG plasmid, the NP helper plasmid, and the L helper plasmid. At 24 hours post-transfection, cells were imaged for mCherry and GPC signal. The scale bar is 20 µM. Images are representative of three independent imaging experiments. (**C**) Quantification of MFI of the mCherry signal (left) and GPC signal (right) for mCherry-expressing cells. One-way ANOVA was performed for statistical analysis. ns, *P* > 0.05; ****, *P* < 0.0001. Data are a pool of three independent imaging experiments with about 250 cells imaged per condition. (**D**) RNA was collected at 24 hpt from cells transfected with wild-type or delVG MGs, and qPCR was performed to measure GPC gene expression. Gene expression was normalized to a housekeeping index of β*-actin* and *GAPDH*. One-way ANOVA was performed for statistical analysis. ****, *P* < 0.0001. Data are representative of three independent experiments with similar results.

We transfected an LCMV wild-type minigenome or minigenomes containing deletions 1560_1613 or 1572_1613 into Vero E6 cells (Fig. 4A). Upon co-transfection with the NP and L proteins, the wild-type minigenome produced strong mCherry and GPC signals while the delVG-containing minigenomes showed a markedly reduced GPC signal, suggesting reduced protein production in the delVG conditions (Fig. 4B). Upon quantification of the mean fluorescent intensity (MFI) of the GPC protein, we found that the delVG minigenomes had significantly lower GPC protein detected for the two deletions tested (Fig. 4C). The decrease in protein production was not due to the decreased replication of the delVG minigenomes. We instead detected slightly increased RNA levels of the viral GPC gene when the IGR-delVGs are present (Fig. 4D). These data suggest that while the IGR-delVG minigenome RNA levels are not lower, the delVG minigenomes produce less GPC compared to the wild type.

One interesting aspect of the S RNA IGR-delVGs is that they produce a frameshift in the GPC open reading frame. This frameshift would add an additional C-terminal tail to the GPC that is about 20 amino acids long, which we termed the C-terminal peptide (Cpep) (Fig. S4A). Even though we observe a decrease in protein production due to the S IGR delVGs during minigenome replication, the novel GPC-Cpep may be produced and play a role in viral interference. To attempt to detect the GPC-Cpep, we developed an antibody against the Cpep motif. As controls for the Cpep antibody, we transfected either the wild-type glycoprotein 2 (GP2) subunit or the GP2-Cpep subunit and confirmed that the Cpep antibody would only recognize the GP2-Cpep by western blot (Fig. S7B) and immunofluorescence (Fig. S7C). We then sought to detect the Cpep in LCMV-infected cells but did not observe the Cpep motif by either western blot (Fig. S7B, right lanes) or immunofluorescence (Fig. S7D). We concluded that the novel GPC open reading frame did not produce enough of the novel protein to be detected during infection. Therefore, the S RNA IGR-delVGs most likely do not produce a novel viral protein that plays a role during viral infection.

### JUNV and PARV produce S RNA IGR-delVGs

**Fig 5:**
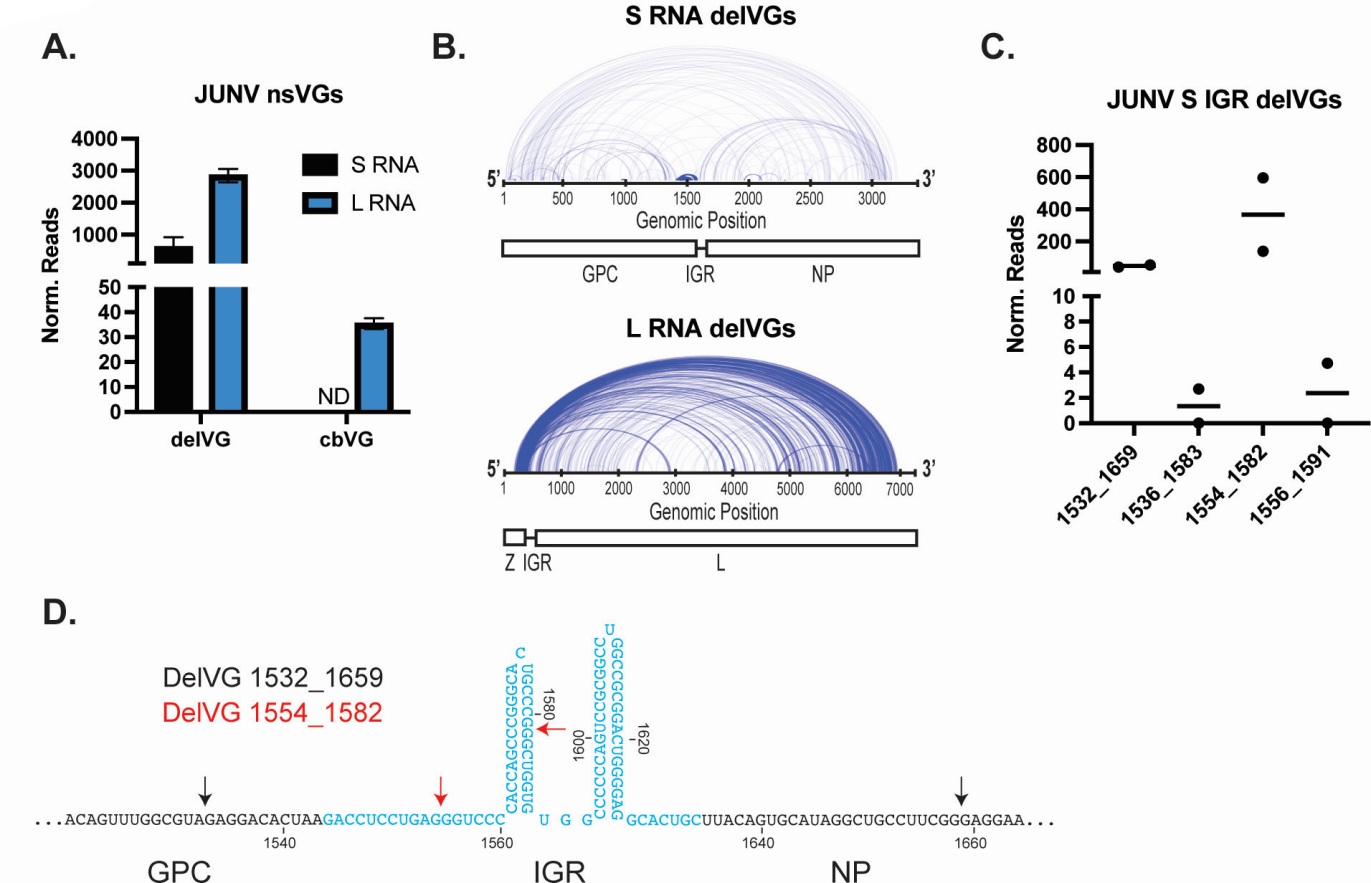
Junin virus produces S RNA IGR-delVGs. Vero E6 cells were infected with Junin virus Candid#1 (JUNV) at an MOI of 0.1. Cellular RNA was isolated at 48 hpi, and RNA sequencing/VODKA2 analysis was performed. (**A**) Quantification of nsVGs during JUNV infection for the S and L RNAs. Reads were normalized as nsVG reads per 1 million viral reads. (B) Arcplot representing the junctions of the JUNV S RNA and L RNA delVGs. Each arc represents an individual delVG species with the width of the arc proportional to the normalized reads detected per delVG species. (**C**) Quantification of the JUNV S RNA IGR-delVGs. (**D**) Schematic representation of the JUNV S RNA IGR-delVGs. Black arrows indicate delVG 1532_1659, and red arrows indicate delVG 1554_1582.

Lastly, we sought to determine if other arenaviruses produced similar IGR-delVGs as LCMV. We infected Vero E6 cells with JUNV Candid#1 or PARV for 48 hours. RNA was collected, sequenced, and analyzed for nsVGs using the VODKA2 analysis pipeline. Both JUNV (Fig. 5A and B) and PARV (Fig. S8A and B) produced delVGs in the S RNA and L RNA, while very few or no cbVGs were detected. JUNV produced four S RNA IGR-delVG species with the most abundant being delVG 1554_1582 (Fig. 5C and D). PARV also produced four species of S RNA IGR-delVGs with delVG 1607_1649 being most detected (Fig. S8C and D). Interestingly, the IGR-delVGs of JUNV and PARV appear to partially remove the IGR, similar to the LCMV delVGs. The locations of the deletion junctions within the IGR stem loops for PARV, JUNV, and LCMV suggest that there may be a conserved mechanism for the generation of these delVGs in arenaviruses.

## DISCUSSION

Here, we show that LCMV produces delVGs that are associated with the inhibition of viral replication. The major species of delVGs are localized to the S RNA IGR and delete out portions of the GPC gene and IGR. Similar delVGs were found in other arenavirus infections, JUNV Candid#1 and PARV. The S RNA IGR-delVGs have increased abundance during higher-interfering LCMV infection, suggesting a mechanism for viral interference. We show that S RNA IGR-delVGs can inhibit the production of viral glycoprotein during LCMV minigenome replication, providing a possible mechanism for the control of infectious particle release.

Important questions remain about how these IGR-delVGs are produced and if the generation of the IGR-delVGs can be modulated. A recent study found that Lassa virus that lacks the nucleoprotein exonuclease activity had increased rates of deletions in both the S RNA and L RNA (31). Interestingly, the major S RNA deletions reported in that study mapped similarly to the IGR region as we observed for LCMV, JUNV, and PARV. It is not understood how the arenavirus NP exonuclease inhibits the formation of the IGR delVGs, but modulation of this activity through small molecule inhibitors could potentially attenuate arenavirus replication by increasing delVG production. Since we observe identical abundant species in separate viral stocks of LCMV-Arm and LCMV-Cl13, we theorize that the S RNA IGR-delVGs can be *de novo*-synthesized in different LCMV infections. We also observe the delVGs in the supernatants of cell infections (Fig. 3; Fig. S2), suggesting that the delVGs can be packaged and transmitted to newly infected cells. The IGR-delVGs occur over a highly structured RNA region, which could cause RNA polymerases to skip and generate delVGs. The generation and regulation of arenavirus delVGs represent an important area of further study for a mechanism of inhibiting viral infections.

The inhibition of GPC protein production by the IGR-delVGs suggests the possibility of targeting the IGRs as an antiviral strategy. Interestingly, the IGR has already been shown to be a good target for attenuating arenaviruses for vaccine generation (14–18). Using reverse genetics, recombinant arenaviruses that contain the S RNA IGR in both the S RNA and L RNA have been developed. These recombinant arenaviruses are attenuated during infection of mice and guinea pigs (17, 18). Since we observe that the S RNA IGR region is a frequent site for delVG generation, it is possible that the L RNA in these recombinant vaccines, which contain the S RNA IGR, have enhanced delVG production, thus preventing protein expression from the L RNA and attenuating the virus. Additionally, other vaccine candidates that show promising attenuation produce truncated viral RNAs (14).

With the rapid development of mRNA vaccines and siRNA-based therapies, utilization of RNA substrates in the clinic is rapidly expanding. IGR-delVGs present an interesting candidate for arenavirus-specific antiviral therapy, which is currently lacking. We observe that minigenomes containing IGR-delVGs can replicate comparably to the full-length minigenome (Fig. 4). We predict that IGR-delVGs delivered to an infected cell would compete with the normal viral genome for the replication machinery, decrease glycoprotein production, and limit infectious particle release resulting in the control of viral infection and spread. Since we observed similar IGR-delVGs with JUNV and PARV, we should be able to develop similar IGR-delVGs for each arenavirus.

While we observe that LCMV IGR-delVGs decrease the production of glycoprotein during minigenome replication, there are limitations to this study. While increased IGR-delVGs associate with viral interference (Fig. 3), whether the IGR-delVGs can inhibit viral replication during infection is still unknown. Further work is needed to determine if IGR-delVGs reduce glycoprotein production in cells and mice infected by LCMV. Additionally, studies are needed to elucidate if delVG inhibition of protein production can occur in other arenavirus infections such as JUNV and PARV.

Overall, we have discovered novel deletions that may present a mechanism for the attenuation of arenavirus replication. IGR-delVGs can inhibit viral protein production during minigenome replication, which in the context of viral replication may allow the virus to control its own replication. These IGR-delVGs present a novel target for the generation of antivirals and provide a mechanism for the attenuation of several arenavirus vaccines.

## MATERIALS AND METHODS

### Cells and viruses

BHK-21 cells (hamster kidney cells, ATCC, CCL-10), Vero E6 cells (green monkey kidney cells, ATCC, CRL-1586), and A549 cells (human lung cells, ATCC, CCL-185) were cultured at 37°C and 5% CO_2_ with Dulbecco’s modified Eagle’s medium (DMEM) (Thermofisher, 11995065) supplemented with 10% fetal bovine serum (FBS), 1 mM sodium pyruvate, 2 mM L-glutamine, and 50 mg/mL gentamicin. All cell lines were treated with a mycoplasma removal agent (MP Biomedicals, 093050044) and routinely tested for mycoplasma before use. LCMV Armstrong 53b (GenBank accession number: L RNA; AY847351.1, S RNA; AY847350.1) and Clone 13 (GenBank accession number: L RNA; DQ361066.1, S RNA; DQ361065.2) were propagated in BHK-21 cells according to published methods (27). Junin virus Candid#1 (GenBank accession number: L RNA; AY819707.2, S RNA; FJ969442.1) and Parana virus (GenBank accession number: L RNA; NC_010761.1, S RNA; NC_010756.1) were propagated in Vero E6 cells.

### LCMV infection

Cells were infected with LCMV Armstrong at indicated MOIs. Briefly, cells were washed with phosphate buffered saline (PBS); then, infectious media containing virus and no FBS was added for 1 hour with rocking at 37°C and 5% CO_2_. Infectious medium was removed, and cell culture medium was added. Infected cells were incubated at 37°C and 5% CO_2_ for indicated timepoints. LCMV was titered by the focus-forming assay. Briefly, Vero E6 cells were infected with sequential dilutions of LCMV supernatant and overlayed with 0.3% agarose with DMEM containing 5% FBS. At 2 days post-infection, cells were fixed with 3.7% formaldehyde, permeabilized with 0.1% Triton X-100, blocked with 5% FBS in PBS, and immunostained for LCMV nucleoprotein. Cells were stained with 1:100 dilution of mouse anti-nucleoprotein (Abcam, AB31774), 1:1,000 dilution of biotinylated goat anti-mouse IgG (Southern Biotech, 1031-08), and 1:1,000 Strep-AP (Invitrogen, 21324). Individual foci were visualized with nitro-blue tetrazolium and 5-bromo-4-chloro-3′-indolyphosphate p-toluidine salt (NBT/BCIP) (Invitrogen, 34042) and quantified.

LCMV-LMP and LCMV-HMP were generated by passaging the LCMV Armstrong stock virus for three passages at MOIs of 0.001 and 1, respectively. Viral supernatants were collected at 72 hours post-infection for each passage. Viral stocks were sequenced by RNA sequencing as described below.

### RNA sequencing and VODKA2 analysis

RNA was collected at indicated timepoints and isolated with Trizol (Thermofisher, 15596026) for cell culture or Trizol LS (Thermofisher, 10296010) for viral supernatants according to the manufacturer’s protocols. RNA integrity and concentration were measured by Bioanalyzer (Agilent) and Qubit (Invitrogen), respectively. Sequencing libraries were prepared with the Illumina Truseq Stranded Total RNA kit with Ribozero Gold Depletion (Illumina, 20020599). Libraries were then sequenced for 2 × 150 paired-end reads on either a NovaSeq 6000 (Illumina) with the WUSTL Genome Technology Access Center or Nextseq 550 (Illumina) with the WUSTL Center for Genome Sciences & Systems Biology.

VODKA2 analysis was performed for the detection of cbVGs and delVGs as previously published (30). The minimal number of bases on each side of a nsVG junction point is 15 bases. Additional filters were added to exclude background from samples. For cbVGs, species with less than two reads and species whose predicted genomes were greater than the wild-type virus genome were excluded from the study. For delVGs, species with less than two reads and species with deletions of five or less nucleotides removed were excluded from the study. Coverage maps of RNA sequencing experiments for each tested RNA genome are shown in Fig. S9.

### Nanopore sequencing

RNA was collected from LCMV Armstrong stocks and utilized for direct RNA sequencing according to the manufacturer’s protocols (Nanopore, SQK-RNA004). Briefly, the genomic S RNA was ligated to nanopore adapters utilizing the following DNA oligos: Oligo A: 5′-/5PHOS/GGCTTCTTCTTGCTCTTAGGTAGTAGGTTC-3′; Oligo B: 5′-GAGGCGAGCGGTCAATTTTCCTAAGAGCAAGAAGAAGCCCGCACAGTGGATCCTAGGCA-3′. After ligation, RNA was sequenced with a Nanopore flowcell (Nanopore, FLO-MIN114) on a MinION Mk1C. Nanopore reads were mapped to the S RNA with minimap2; then, coverage and deleted residues were quantified with Samtools mpileup.

### RNAscope

Vero E6 cells were either transfected with pCAGGs-GPC-HA or pCAGGs-GPC-Cpep-HA or infected with LCMV-LMP or LCMV-HMP stocks at an MOI of 0.1. At indicated timepoints, cells were fixed with 3.7% formaldehyde, then processed for imaging using the RNAScope Multiplex Fluorescent Detection Kit v2 (ACD Bio, 323110) according to the manufacturer’s protocol. The GPC gene probe detects nucleotides 201–243 of the S RNA segment. The DelVG 1572-1612 probe detects nucleotides 1542–1572 and 1612–1623 of the S RNA. Probes were designed by ACD Bio (acdbio.com). The dyes used for detection were the TSA Plus Cy3 System (Akoya Biosciences, NEL744001KT) and TSA Plus Cy5 System (Akoya Biosciences, NEL745001KT). Coverslips were mounted with ProLong Diamond Antifade (Thermofisher, P36961) and imaged using a Zeiss Axio Observer Widefield microscope (Zeiss).

For quantification, approximately 1,000 cells and 200 infected cells were imaged per individual replicate. Images were quantified using Aggrecount automated image analysis (https://aggrecount.github.io) using Fiji software (https://imagej.net/software/fiji/). In brief, cells were counted as infected if they had cell mean fluorescence over the threshold for the GPC probes. Cells were counted as delVG positive if they had cell mean fluorescence over the threshold for the delVG probes.

### RT-PCR and qPCR

RNA was collected at indicated timepoints and isolated with Trizol (Thermofisher, 15596026) for cell culture or Trizol LS (Thermofisher, 10296010) for viral supernatants according to the manufacturer’s protocols. cDNA was generated with random hexamers using the High-Capacity RNA to cDNA kit (Thermofisher, 4387406) according to the manufacturer’s protocols. PCRs were performed with Hot Start Taq DNA Polymerase (NEB, M0495L). IGR delVG bands were separated on a 2% agarose gel. For qPCR quantification, cDNA was quantified with Power SYBR Green Mix (Thermofisher, 4367660). For the quantification of delVG 1572_1613, Taqman Universal Master Mix (Thermofisher, 4440040) was used to amplify the cDNA generated above. All qPCRs were performed on a Quantstudio 5 PCR system (Thermofisher, A34322). SYBR Green qPCRs were normalized to a housekeeping index calculated from the expression of GAPDH and beta-actin genes. Taqman assays were performed with primers and probes in the same PCR reaction. Taqman assays were normalized to the GPC gene. All primer sets and probes have tested efficiencies above 95%. The sequences for primers used are shown in Table S6.

### Minigenome replicon system

The LCMV S RNA minigenome plasmid and LCMV protein expression plasmids were kindly gifted by Dr. Luis Martínez-Sobrido (UTMB) (15). The S RNA MG used was cloned to include a human PolI promoter, the full-length GPC, and an mCherry gene replacing the NP gene. The plasmid was digested with AvrII (NEB, R0174S), then gene fragments were inserted with NEBuilder HIFI DNA Assembly Mix (NEB, E5520S). For transfection and replication, 293T cells were co-transfected with Minigenome, NP, and L plasmids with Lipofectamine 2000 (Thermofisher, 11668019). Cells were incubated for 24 hours until imaged for mCherry (Abcam, AB213511) and GPC expression (GP2 antibody, 13.6, Martínez-Sobrido Lab) or processed for qPCR. The monoclonal Cpep-antibody (5G5) was developed by the Washington University Hybridoma Core.

### *In vitro* transcription of minigenome RNA

The LCMV S RNA minigenome was cloned into a pSL1180-T7 plasmid. *In vitro* transcription was performed with the Megascript T7 Transcription Kit (Thermofisher, AM1334), and RNA was isolated with LiCl precipitation according to the manufacturer’s protocols. Purified RNA was measured by Qubit and tested for quality by Bioanalyzer (Agilent).

### Statistics

All statistics were calculated using GraphPad Prism Version 9. Specific tests and significance values are indicated in each figure legend.

## Data Availability

All data are available in the main text or the supplemental materials. Raw sequencing files are deposited at NCBI Sequence Read Archive (SRA) with the BioProject ID PRJNA1032960.

## SUPPLEMENTAL MATERIAL

Supplemental material mbio.01612-24-s0001.pdf Supplemental figures and captions for Tables S1 to S6.

Supplemental tables mbio.01612-24-s0002.xlsx Tables S1 to S3.

Table S4 mbio.01612-24-s0003.xlsx S RNA IGR-delVG species.

Table S5 mbio.01612-24-s0004.xlsx Sanger sequencing of S RNA IGR-delVG RT-PCR bands.

Table S6 mbio.01612-24-s0005.xlsx PCR primers.

ASM does not own the copyrights to Supplemental Material that may be linked to, or accessed through, an article. The authors have granted ASM a non-exclusive, world-wide license to publish the Supplemental Material files. Please contact the corresponding author directly for reuse.
