# Supplementary material for: Characterization of non-standard viral genomes during arenavirus infections identifies prominent S RNA intergenic region deletions
Source: mBio. 2024 Sep 11;15(10):e01612-24. doi: 10.1128/mbio.01612-24 (PMC11481572; doi:10.1128/mbio.01612-24)
Supplement: Supplemental material — Supplemental figures and captions for Tables S1 to S6. [file mbio.01612-24-s0001.pdf]

SUPPLEMENTAL MATERIALS

Table S1: Summary of VODKA2 Quantification of Individual nsVGs

Table S2: VODKA2 detected S RNA nsVGs

Table S3: VODKA2 detected L RNA nsVGs

Table S4: S RNA IGR-delVG Species

Table S5: Sanger Sequencing of S RNA IGR-delVG RT-PCR Bands

Table S6: PCR Primers

Figure S1: High MOI LCMV infections do not induce cell death. A549 cells were infected with LCMV-Arm at indicated MOIs. At 48 hpi, cells were measured for cell death with either A) Cell Titer Glo assay measuring luciferase activity for cellular ATP levels or B) LDH Assay measuring the release of LDH into the cellular media. One-way ANOVA was performed. ns: $p>0.05$. C) Diagram of primer and probes for the delVG Taqman RT-PCR assay. D) A549 cells were infected with LCMV-Arm at indicated MOIs. At 48 hpi, cellular RNA was collected and the delVG Taqman assay was performed. Data was normalized to the GPC probe. One-way ANOVA was performed. ns: $p>0.05$; *: $p<0.05$; ***: $p<0.001$.

Figure S2: Distribution of nsVGs during an LCMV infection timecourse. A549 cells were infected with LCMV-Arm at an MOI of 0.1. At 12, 24, and 48 hpi, cellular and supernatant RNA was collected, sequenced, and analyzed with the VODKA2 pipeline. A) Representation of Break and Rejoin points of individual S RNA cbVGs at 48 hpi from cellular RNA. B) Representation of Break and Rejoin points of individual L RNA cbVGs at 48 hpi from cellular RNA. 5' UTR-containing cbVGs are indicated in red. 3' UTR-containing cbVGs are indicated in blue. Reads are normalized as nsVG reads per 1 million viral reads. C) Quantification of S RNA delVGs and cbVGs at indicated timepoints for cellular and supernatant RNA. Data is the mean of R1 and R2 reads

processed separately. D) Quantification of L RNA delVGs and cbVGs at indicated timepoints for cellular and supernatant RNA. Data is the mean of R1 and R2 reads processed separately.

Figure S3. LCMV-Cl13 predominately produces S RNA IGR delVGs. A549 cells were infected with LCMV Clone 13 at an MOI of 0.1. RNA was collected at 48 hpi, sequenced, and analyzed by the VODKA2 pipeline. A) Quantification of nsVG species for the S RNA (red) and L RNA (blue). B) Quantification of the S RNA IGR-delVGs. Data is representative of 3 biological replicates. Reads are normalized as nsVG reads per 1 million viral reads.

Figure S4: Confirmation of LCMV S RNA delVGs A) A549 cells were infected with LCMV Armstrong at an MOI of 0.1. At 48 hpi, RNA was collected and RT-PCR was performed. Bands are indicated for the S IGR delVG and genomic S RNA. B) Bioanalyzer of *in vitro* transcribed and purified LCMV minigenome RNA. C) Representation of 5' junction and 3' junction points of individual *in vitro*-transcribed minigenome delVGs. Each arc represents an individual delVG species with the width of the arc proportional to the normalized reads detected per delVG species. X axis is the nucleotide position of the S RNA minigenome genome. D+E) LCMV supernatant RNA was collected and Nanopore sequencing was performed for the genomic-sense of the S RNA. D) Coverage of Nanopore reads mapped to the S RNA genome of LCMV-Arm. E) The percentage of bases deleted at each individual genomic position was quantified. Data shown represents the entire S RNA (top) and a zoom-in of the S RNA IGR (bottom).

Figure S5: LCMV-HMP infection has increased IGR-delVGs at 24 hpi but does not activate early IFN pathways. A549 cells were infected with LCMV-LMP or LCMV-HMP at an MOI of 1 and at indicated timepoints RNA was collected and qPCR was performed for A) viral S RNA, B) IFNB1, or C) IL-29. Gene expression is normalized to *ß-Actin* and *GAPDH*. *: $p<0.05$; **: $p<0.01$; ***: $p< 0.001$; ****: $p<0.0001$. D) qPCR was performed to detect delVG 1572_1613 or GPC gene.

DelVG 1572_1613 was normalized to GPC gene. Two-way ANOVA was performed. ns: p>0.05; *: p<0.05.

Figure S6: LCMV-HMP stock has increased abundance of S RNA IGR-delVGs. RNA was collected from LCMV-LMP or LCMV-HMP stocks, sequenced, and analyzed with the VODKA2 pipeline. A) Quantification of nsVGs in LCMV Std MOI or LCMV High MOI stocks. 2-way ANOVA was performed. ns: p>0.05; ****: p<0.0001 B) Quantification of individual S RNA IGR-delVGs in LCMV Std MOI or LCMV High MOI stocks. 2-way ANOVA was performed. ns: p>0.05; **: p<0.01; ***: p<0.001.

Figure S7: S RNA delVGs do not produce an abundant novel GPC protein. A) Translation of the C-terminal end of the GPC open reading frame for the wild-type (WT) S RNA, delVG 1560_1613, and delVG 1572_1613. B) Vero E6 cells were either transfected with HA-GP2 or HA-GP2-Cpep or infected with LCMV-LMP or LCMV-HMP at an MOI of 0.1. Cellular protein lysate was collected after 48 hours. Western blots were performed and stained for the C-terminal peptide (Cpep), GP2 subunit, HA-tag, and Alpha-tubulin. C) Immunofluorescence of Vero E6 cells transfected with HA-GP2 or HA-GP2-Cpep with an HA-tag antibody (green) and a Cpep antibody (magenta). D) Immunofluorescence of Vero E6 cells mock-infected or LCMV-High MOI-infected at an MOI of 1. Cells were fixed at 48 hpi and stained with NP antibody (magenta) and the Cpep antibody (green).

Figure S8: Parana Virus produces S RNA delVGs. Vero E6 cells were infected with Parana Virus (PARV) at an MOI of 0.1. Cellular RNA was isolated at 48 hpi and RNA-sequencing/VODKA2 analysis was performed. Reads were normalized as nsVG reads per 1 million viral reads. A) Quantification of nsVGs during PARV infection for the S and L RNAs. B)

Arcplot representing the junctions of the PARV S RNA and L RNA delVgs. Each arc represents an individual delVG species with the width of the arc proportional to the normalized reads detected per delVG species. C) Quantification of the PARV S RNA IGR-delVGs D) Schematic representation of the PARV S RNA IGR-delVGs. Black arrows indicate delVG 1607_1649. Data are representative of 2 individual sequencing experiments.

Figure S9: Coverage maps of RNA-Sequencing Experiments. A) Coverage maps of the S RNA(Left) and L RNA (Right) of LCMV-Arm infections from the RNA-Sequencing data in Figure 1E-I. B) Coverage maps of RNA-sequencing of IVT LCMV-MG infections from the RNA-Sequencing data in Figure S4B-C. C) Coverage maps of the S RNA(Left) and L RNA (Right) of JUNV-C1 infections from the RNA-Sequencing data in Figure 5. D) Coverage maps of the S RNA(Left) and L RNA (Right) of PARV infection from the RNA-Sequencing data in Figure 5.

Fig S1

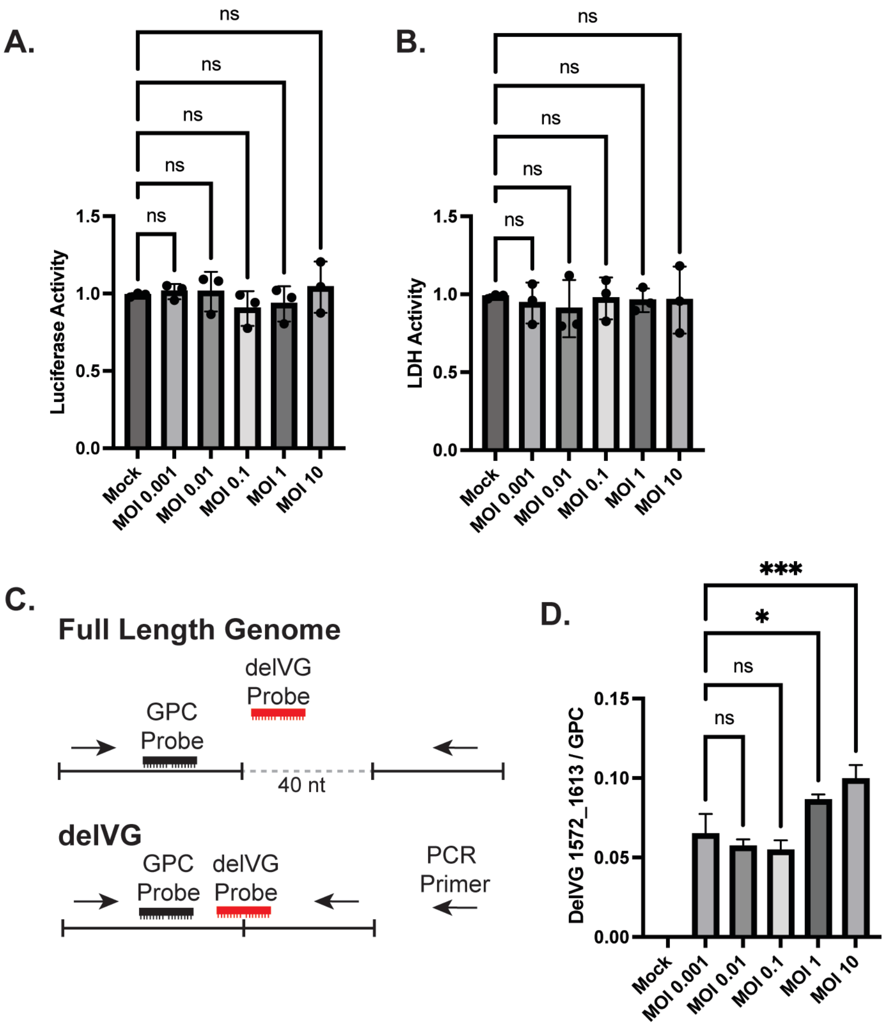

A.
ns
1.5
1.0
0.5
0.0
Luciferase Activity
Mock
MOI 0.001
MOI 0.01
MOI 0.1
MOI 1
MOI 10
B.
LDH Activity
C.
Full Length Genome
delVG
Probe
GPC
Probe
40 nt
delVG
PCR
Primer
D.
***
*
0.15
0.10
0.05
0.00
DelVG 1572_1613 / GPC

Fig S2

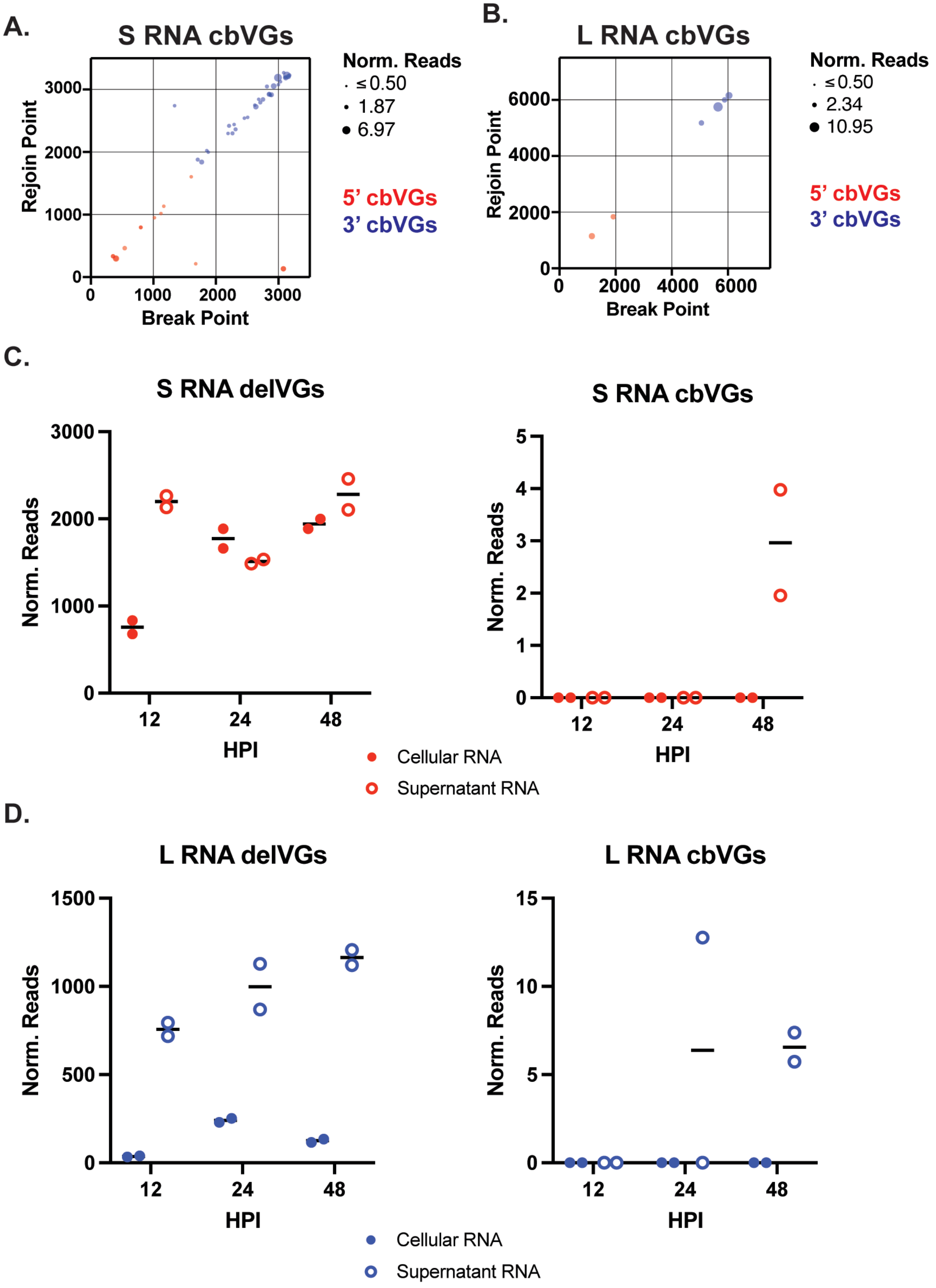
A.
S RNA cbVGs
Rejoin Point
Break Point
Norm. Reads
≤0.50
1.87
6.97
5' cbVGs
3' cbVGs
B.
L RNA cbVGs
Rejoin Point
Break Point
Norm. Reads
≤0.50
2.34
10.95
5' cbVGs
3' cbVGs
C.
S RNA delVGs
S RNA cbVGs
Norm. Reads
HPI
Cellular RNA
Supernatant RNA
D.
L RNA delVGs
L RNA cbVGs
Norm. Reads
HPI
Cellular RNA
Supernatant RNA

Fig S3

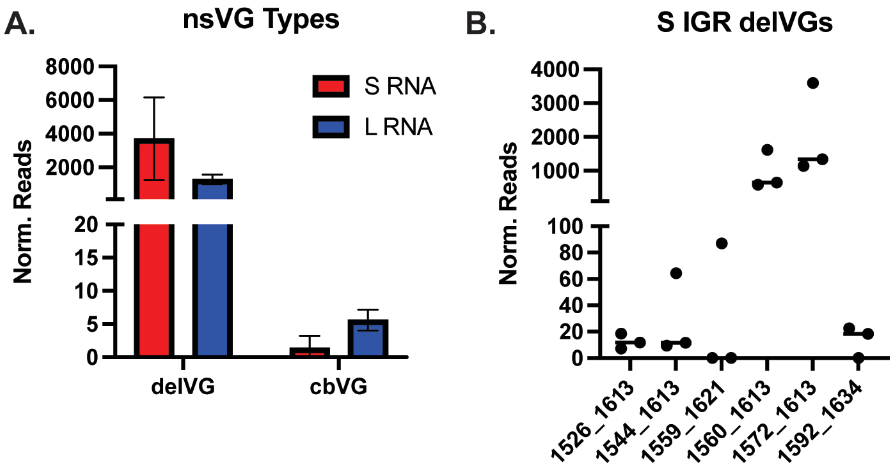
A.
nsVG Types
8000
6000
4000
2000
20
15
10
5
0
Norm. Reads
S RNA
L RNA
delVG
cbVG
B.
S IGR delVGs
4000
3000
2000
1000
100
80
60
40
20
0
Norm. Reads
1526_1613
1544_1613
1559_1621
1560_1613
1572_1613
1592_1634

Fig S4

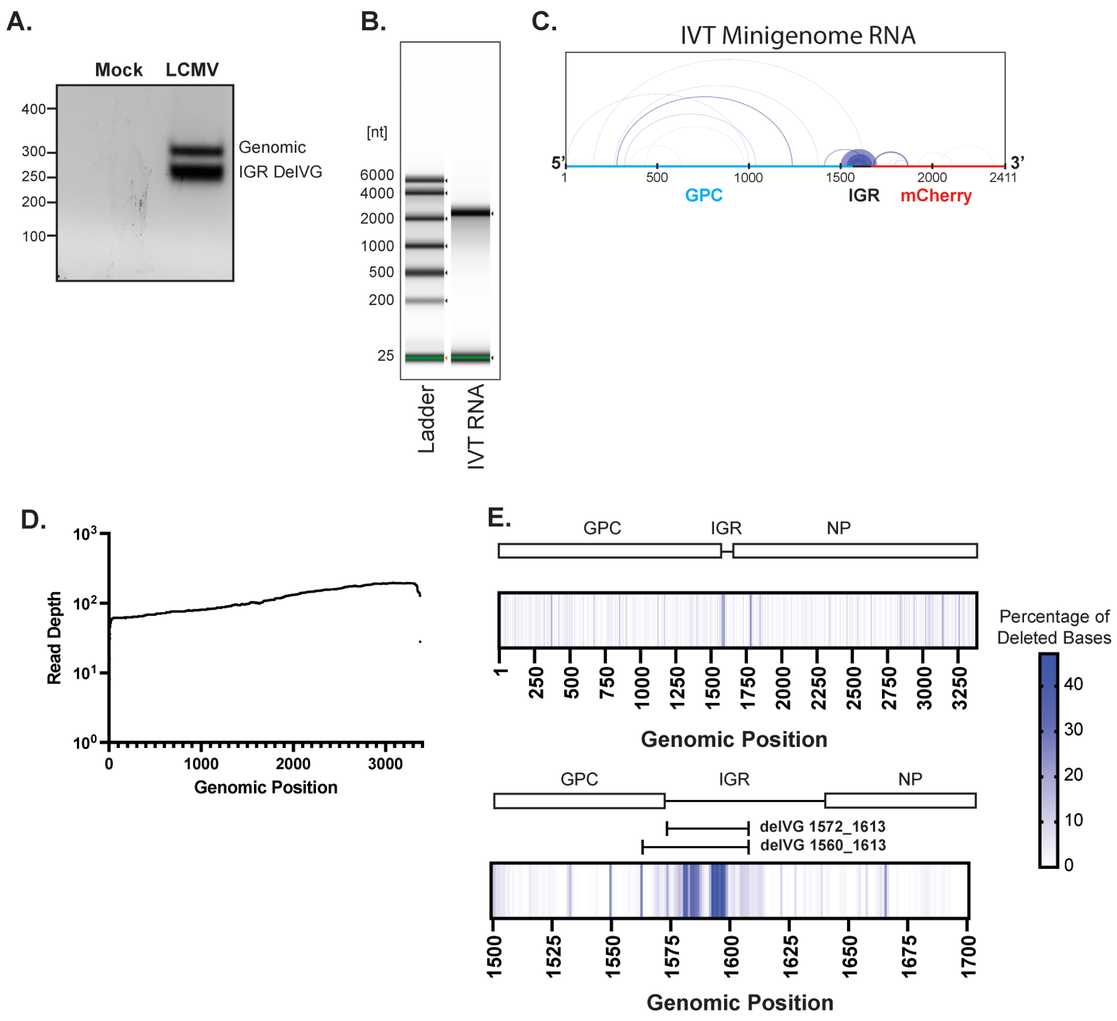

A.
Mock
LCMV
400
300
250
200
100
Genomic
IGR DelVG
B.
[nt]
6000
4000
2000
1000
500
200
25
Ladder
IVT RNA
C.
IVT Minigenome RNA
5'
3'
1
500
1000
1500
2000
2411
GPC
IGR
mCherry
D.
Read Depth
Genomic Position
E.
GPC
IGR
NP
Percentage of Deleted Bases
Genomic Position
delVG 1572_1613
delVG 1560_1613
Genomic Position

Fig S5

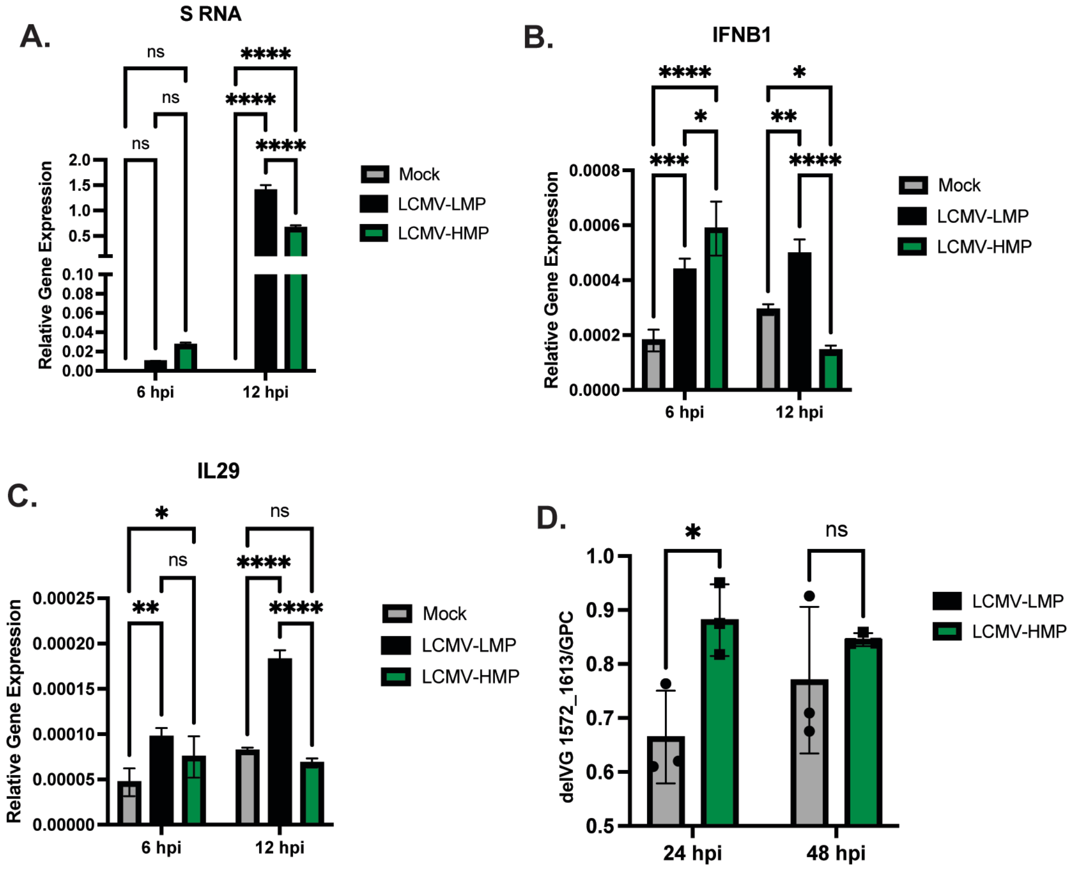
A.
S RNA
Relative Gene Expression
6 hpi
12 hpi
ns
****
Mock
LCMV-LMP
LCMV-HMP
B.
IFNB1
Relative Gene Expression
6 hpi
12 hpi
****
***
**
*
C.
IL29
Relative Gene Expression
6 hpi
12 hpi
D.
delVG 1572_1613/GPC
24 hpi
48 hpi
LCMV-LMP
LCMV-HMP

Fig S6

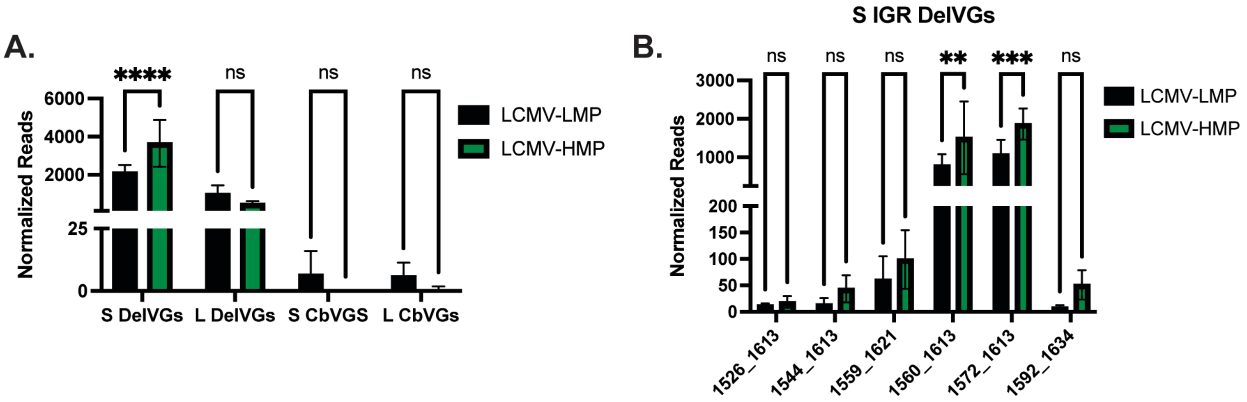

A.
****
ns
ns
ns
6000
4000
2000
25
0
Normalized Reads
S DelVGs
L DelVGs
S CbVGS
L CbVGs
LCMV-LMP
LCMV-HMP
B.
S IGR DelVGs
ns
ns
ns
**
***
ns
3000
2000
1000
200
150
100
50
0
Normalized Reads
1526_1613
1544_1613
1559_1621
1560_1613
1572_1613
1592_1634
LCMV-LMP
LCMV-HMP

Fig S7

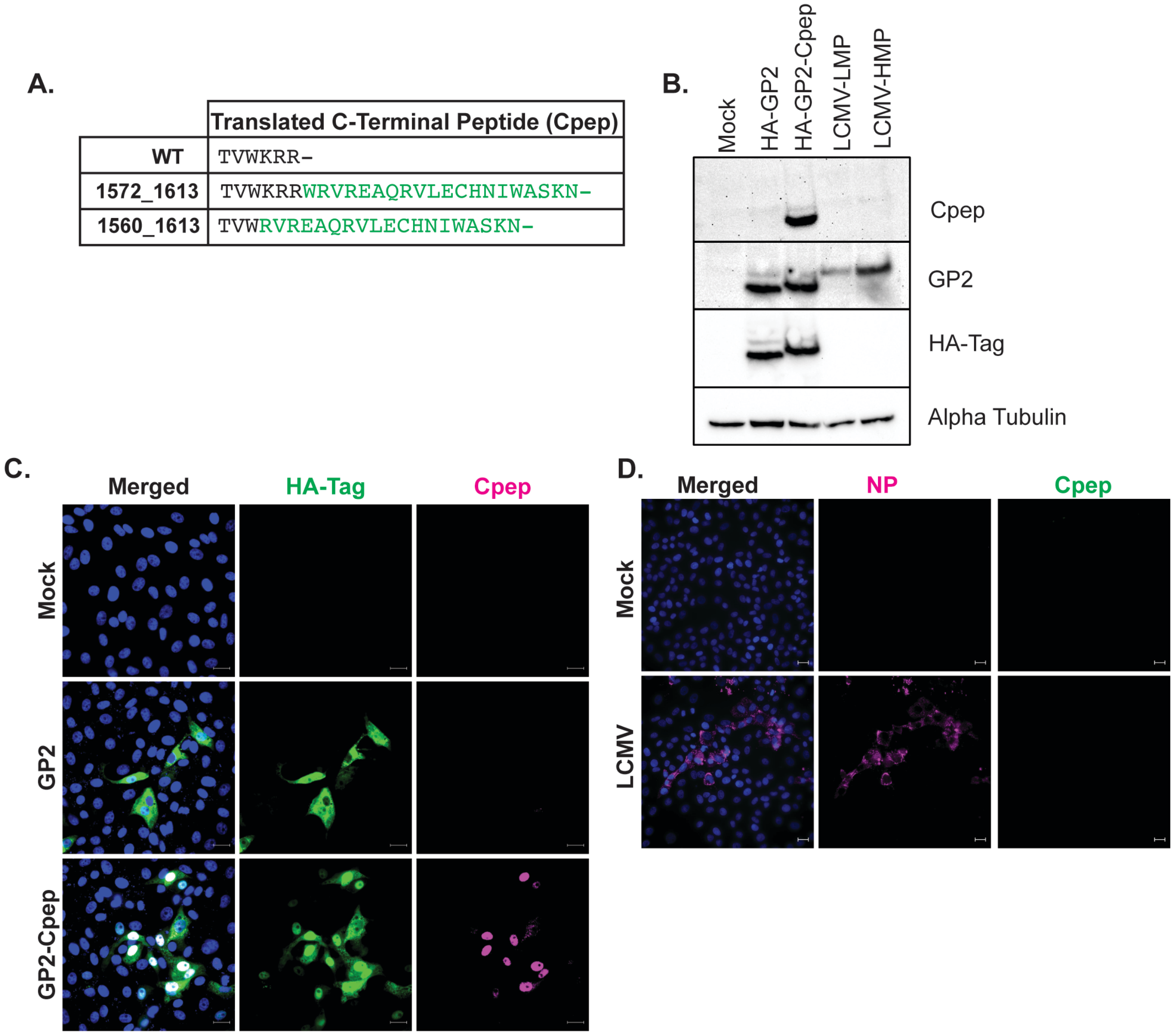
A.
Translated C-Terminal Peptide (Cpep)
WT
TVWKRR-
1572_1613
TVWKRRWRVREAQRVLECHNIWASKN-
1560_1613
TVWRVREAQRVLECHNIWASKN-
B.
Mock
HA-GP2
HA-GP2-Cpep
LCMV-LMP
LCMV-HMP
Cpep
GP2
HA-Tag
Alpha Tubulin
C.
Merged
HA-Tag
Cpep
Mock
GP2
GP2-Cpep
D.
Merged
NP
Cpep
Mock
LCMV

Fig S8

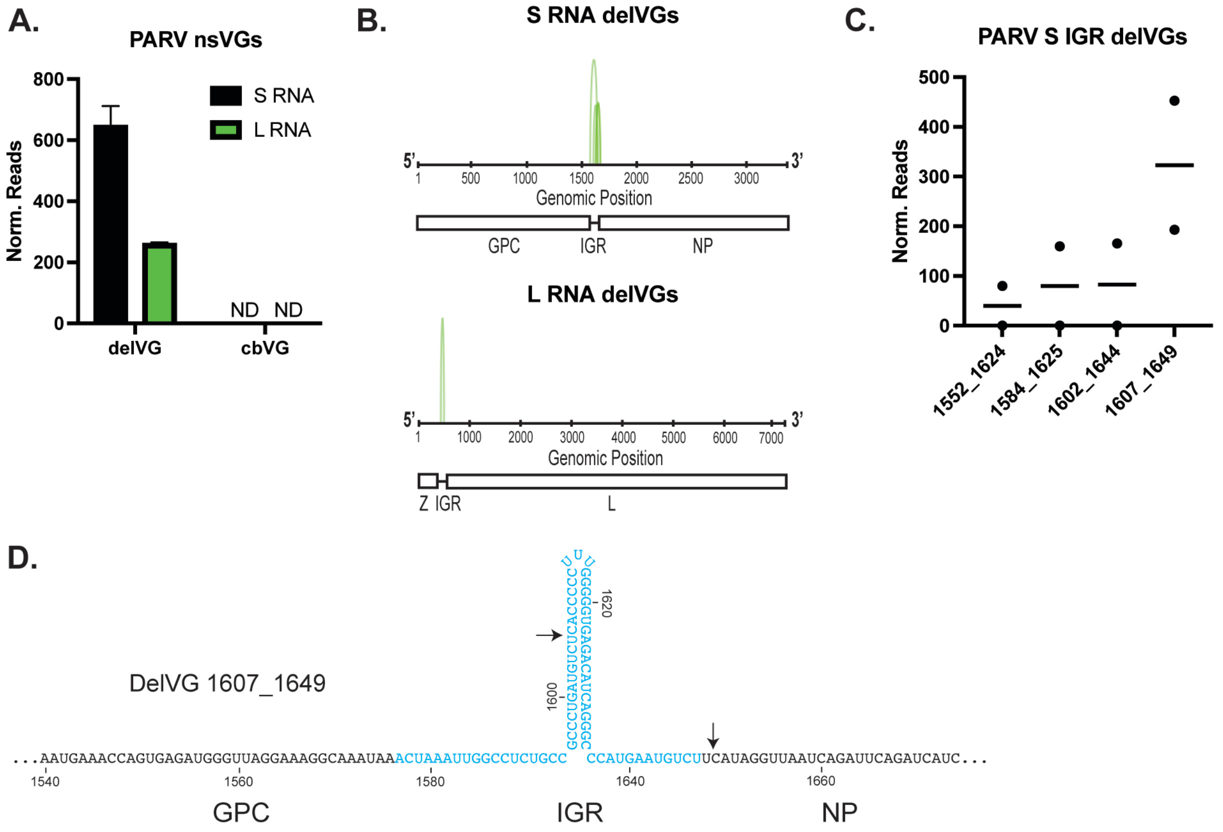
A.
PARV nsVGs
S RNA
L RNA
Norm. Reads
800
600
400
200
0
ND
ND
delVG
cbVG
B.
S RNA delVGs
5'
3'
1
500
1000
1500
2000
2500
3000
Genomic Position
GPC
IGR
NP
L RNA delVGs
5'
3'
1
1000
2000
3000
4000
5000
6000
7000
Genomic Position
Z
IGR
L
C.
PARV S IGR delVGs
Norm. Reads
500
400
300
200
100
0
1552_1624
1584_1625
1602_1644
1607_1649
D.
DelVG 1607_1649
1600
1620
...AAUGAAACCAGUGAGAUGGGUUAGGAAAGGCAAAUAAACUAAAUUGGCCUCUGCC CCAUGAAUGUCUUCAUAGGUUAAUCAGAUUCAGAUCAUC...
1540
1560
1580
1640
1660
GPC
IGR
NP

Fig S9

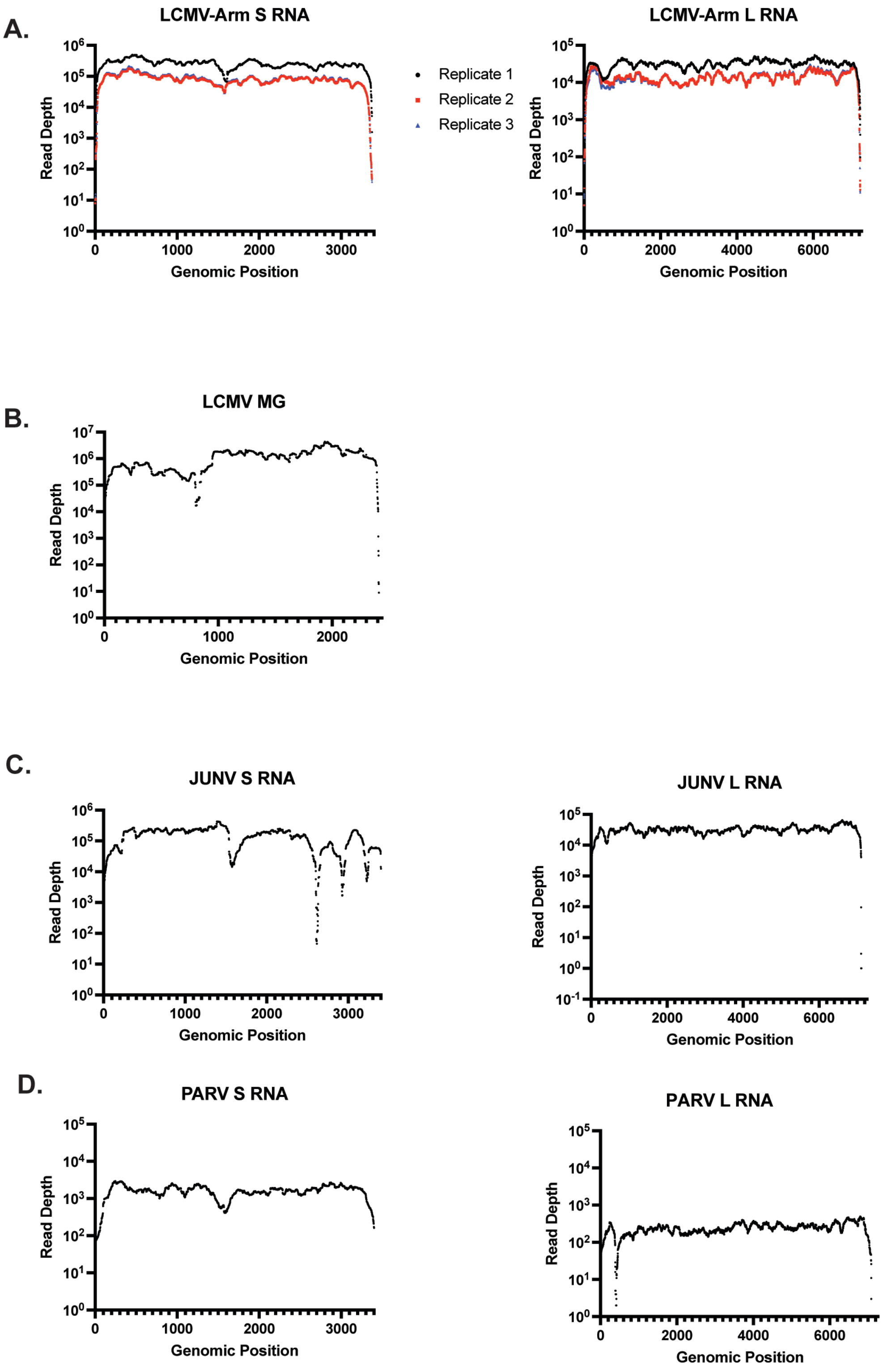
A.
LCMV-Arm S RNA
LCMV-Arm L RNA
Replicate 1
Replicate 2
Replicate 3
Read Depth
Genomic Position
B.
LCMV MG
Read Depth
Genomic Position
C.
JUNV S RNA
JUNV L RNA
Read Depth
Genomic Position
D.
PARV S RNA
PARV L RNA
Read Depth
Genomic Position
